# Pancreatic Ductal Adenocarcinoma: The Dawn of the Era of Nuclear Medicine?

**DOI:** 10.3390/ijms22126413

**Published:** 2021-06-15

**Authors:** Christopher Montemagno, Shamir Cassim, Nicolas De Leiris, Jérôme Durivault, Marc Faraggi, Gilles Pagès

**Affiliations:** 1 Département de Biologie Médicale, Centre Scientifique de Monaco, 98000 Monaco, Monaco; shamir_cassim@yahoo.fr (S.C.); jdurivault@centrescientifique.mc (J.D.); gilles.pages@unice.fr (G.P.); 2 Institute for Research on Cancer and Aging of Nice, Centre Antoine Lacassagne, CNRS UMR 7284 and IN-SERM U1081, Université Cote d’Azur, 06200 Nice, France; 3 LIA ROPSE, Laboratoire International Associé Université Côte d’Azur—Centre Scientifique de Monaco, 98000 Monaco, Monaco; 4 Nuclear Medicine Department, Grenoble-Alpes University Hospital, 38000 Grenoble, France; NDeleiris@chu-grenoble.fr; 5 Laboratoire Radiopharmaceutiques Biocliniques, Univ. Grenoble Alpes, INSERM, CHU Grenoble Alpes, 38000 Grenoble, France; 6 Centre Hospitalier Princesse Grace, Nuclear Medicine Department, 98000 Monaco, Monaco; Marc.FARAGGI@chpg.mc

**Keywords:** PDAC, nuclear medicine, molecular imaging, internal vectorized radiotherapy

## Abstract

Pancreatic ductal adenocarcinoma (PDAC), accounting for 90–95% of all pancreatic tumors, is a highly devastating disease associated with poor prognosis. The lack of accurate diagnostic tests and failure of conventional therapies contribute to this pejorative issue. Over the last decade, the advent of theranostics in nuclear medicine has opened great opportunities for the diagnosis and treatment of several solid tumors. Several radiotracers dedicated to PDAC imaging or internal vectorized radiotherapy have been developed and some of them are currently under clinical consideration. The functional information provided by Positron Emission Tomography (PET) or Single Photon Emission Computed Tomography (SPECT) could indeed provide an additive diagnostic value and thus help in the selection of patients for targeted therapies. Moreover, the therapeutic potential of β^-^- and α-emitter-radiolabeled agents could also overcome the resistance to conventional therapies. This review summarizes the current knowledge concerning the recent developments in the nuclear medicine field for the management of PDAC patients.

## 1. Introduction

Pancreatic ductal adenocarcinoma (PDAC) is by far the most common type of pancreatic neoplastic disease, accounting for about 90 to 95% of all pancreatic malignancies [1,2]. PDAC is an aggressive disease, representing the fourth most frequent cause of cancer-related deaths worldwide in 2018 [3,4]. The incidence of PDAC is increasing and PDAC is predicted to become the second deadliest malignancy by the year 2030. Indeed, a two-fold augmentation of PDAC cases is expected for the next ten years [5,6]. During the last decades, significant improvements have been achieved in the screening and treatment of different solid tumors, incrementing the patient’s chance to be cured. Nevertheless, insignificant modification of the mortality-to-incidence ratio has been evidenced in PDAC. PDAC has the shortest 5-year overall survival (OS) rate of all major cancers (7%) [7]. Efficacy of treatments and outcome of PDAC are largely determined by the disease stage at the diagnosis, but unfortunately PDAC patients are usually diagnosed at advanced stages, limiting the therapeutic opportunities [7]. The only curative therapy available is surgical resection followed by adjuvant therapy [8]. Unfortunately, most of the patients (80–90%) with PDAC remains asymptomatic until the disease develops to advanced stages or metastatic disease that make them ineligible to surgery [9], explaining the poor prognosis [10]. During the last 30 years, the 5-year survival of surgically resected patients has increased from 5 up to 54% [11,12,13]. However, the 5-year survival remains unchanged over the same period in unresected PDAC patients [10,14].

Differences in the molecular biology of PDAC may contribute to its early metastatic dissemination. Indeed, pre-clinical models of PDAC indicate that metastases can be detected even in the absence of any pancreatic primary tumor [15]. PDAC is therefore suggested to be a systemic pathology and multidisciplinary management of this disease becomes of great of importance. Therefore, the identification of novel therapeutic targets and the modalities to improve clinical management of the disease and life expectancy of PDAC patients are urgently needed. The diagnosis and therapeutic potential of nuclear medicine could provide new opportunities for the management of PDAC patients. Nuclear medicine indeed came up with radiopharmaceuticals that impart the ability to destroy tumor cells specifically with high-energy-emitting radionuclides [16,17]. The emergence and advent of theranostics, a combination of a single drug used for both diagnosis and therapeutic purposes, has now opened a new avenue in the field of personalized treatments. In recent years, theranostics has been successfully applied to a whole range of malignancies, including neuroendocrine tumors and prostate cancer [18,19]. In this review, we first discuss the different available molecules used in the clinic for PDAC imaging and staging, to finally emphasize the different imaging and theranostic agents that are currently under development for the management of PDAC, including radioligand therapy. 

## 2. Radiotracers Clinically Available for PDAC Diagnosis, Staging and Monitoring

### 2.1. ^18^F-FDG 

Diagnosis of suspected PDAC is based on clinical suspicion and a combination of imaging modalities, including computed tomography (CT), magnetic resonance imaging (MRI) and endoscopic ultrasound (US) [20]. Unfortunately, these imaging modalities are of limited value in the detection of small primary tumors and small-disseminated masses. Over the past two decades, the potential of positron emission tomography (PET) imaging using 18-fluorodeoxyglucose (^18^F-FDG) has been investigated for the diagnosis, staging and detection of recurrence of PDAC. ^18^F-FDG PET takes advantage of the Warburg effect, a hallmark of cancer in which proliferating tumor cells produce energy via glycolysis at higher rates as compared to normal tissues [21,22,23]. ^18^F-FDG PET is a sensitive imaging modality for detection, staging and response assessment in oncology in most solid tumors [24]. FDG, a glucose analogue, is transported into the cells via glucose transporters, phosphorylated by hexokinase, but does not go further into the glycolysis steps, thereby allowing its accumulation into the cells. Almost 90% of PDAC exhibit mutation of the K-Ras oncogene, promoting glucose uptake through the upregulation of hexokinase-2 or glucose transporters [25,26]. ^18^F-FDG uptake was investigated in mice models of PDAC and demonstrated an increased uptake throughout disease progression from pancreatic intraepithelial neoplasia precursor lesions to PDAC [27]. ^18^F-FDG PET has consequently been proposed as a relevant diagnosis modality for PDAC. Nevertheless, the role of ^18^F-FDG PET in the early detection of PDAC still remains controversial. Some guidelines indeed suggest that ^18^F-FDG PET has a very limited role in the diagnosis of PDAC whereas other studies reported the augmented ability of ^18^F-FDG PET in PDAC detection, over CT and MRI [28,29]. A systematic review of 54 studies recently reported that, while ^18^F-FDG PET had a superior sensitivity when compared to CT and MRI, the specificity of CT and MRI is higher [30]. However, ^18^F-FDG PET failed to distinguish PDAC from focal mass-forming pancreatitis, which was reported to be ^18^F-FDG positive in nearly 80% of cases [31]. Importantly, the most important advantages of ^18^F-FDG PET over CT and MRI are (1) it detects distant and potentially unknown metastases by performing a whole-body scan; and (2) it monitors early tumor responses [32]. As for other solid tumors, metabolic changes in PET precede anatomic changes in tumor size [33]. CT cannot detect small tumors [34]. Hence, following chemo/radiotherapy, ^18^F-FDG-PET more accurately evaluates the treatment responses compared to CT. A recent study on 22 patients showed that the response to neoadjuvant therapy in PDAC was more efficiently measured as compared to CT [35]. Several studies demonstrated the role of PET imaging in monitoring tumor recurrence. For instance, Sperti et al. showed that PET could detect earlier tumor relapse in comparison to CT, with a diagnostic accuracy of 96% and 57%, respectively [36]. Wang et al. recently confirmed these findings using ^18^F-FDG in resected PDAC [37]. ^18^F-FDG PET imaging has the ability to distinguish treatment-related fibrosis and inflammation from residual or progressive tumors, as observed by Javery et al. in a cohort of 49 patients [38]. Besides, pre-operative PET imaging predicts tumor recurrence and prognosis in PDAC patients [39]. Indeed, the high standardized uptake value (SUVmax) of patients with PDAC, which is the most common PET parameter used in the clinic, was associated with worse overall survival (OS) and progression-free survival (PFS) [29,40,41]. The emergence of a PET/MRI hybrid system and of radiomics analysis should provide added value in the management of patients with PDAC.

### 2.2. ^18^F-FLT 

3-Deoxy-3-18F-fluorothymidine (^18^F-FLT) is a fluorinated tracer proposed as an imaging biomarker of cell proliferation. ^18^F-FLT is phosphorylated by thymidine-kinase-1 during the S phase and trapped inside the cells, providing an indirect measure of proliferation [42]. Pre-clinical studies carried out in mice demonstrated the ability of ^18^F-FLT to non-invasively image PDAC cells [43]. The role of ^18^F-FLT in the clinic has been investigated in several studies. ^18^F-FLT was found to be negative in 10 benign pancreatic lesions but 15/21 of malignant tumors display high uptake of ^18^F-FLT, thereby suggesting a significant diagnostic potential [44]. Nevertheless, conclusions regarding the use of ^18^F-FLT remain controversial. Quon et al. indeed reported a poor detectability of lesions and a decreased uptake level in the primary tumor foci [45]. Despite its poor sensitivity, a recent study however showed that initial ^18^F-FLT activity is correlated to poor outcomes in PDAC patients [46]. One limitation of this study is certainly the use of a stand-alone PET without any simultaneous CT acquisition. The use of hybrid PET/CT or PET/MRI may thus lead to a greater accuracy. Clinical investigations should be conducted by a dual-image modality and in a higher number of patients to conclude about the real potential role of ^18^F-FLT in managing patients with PDAC. 

### 2.3. ^18^F-FMISO and ^18^F-FAZA

Hypoxia, a condition of low oxygen availability, is a common feature of solid tumors affecting many aspects of tumor biology and promoting resistance to therapy [47]. PDAC is a poorly vascularized tumor and is characterized by a strong desmoplastic reaction, which contributes to the hypoxic state. A poor blood supply is associated with poor prognosis in PDAC patients [48]. In a constitutive hypoxic microenvironment, PDAC constitutively express the hypoxia-inducible factor-1 (HIF-1). HIF-1 expression levels are associated with tumor progression and metastasis [49,50]. The hypoxic environment of PDAC thus provides the rational for imaging hypoxia and investigating its role in the management of patients. Two radiotracers, ^18^F-fluoromisonidazole (^18^F-FMISO) and ^18^F-fluoroazomycin arabinoside (^18^F-FAZA), have been evaluated in PDAC. However, their tumor uptakes were found to be weak, thereby limiting their interest in the imaging of PDAC [51,52]. However, a recent study carried out on 20 PDAC patients prior to surgery showed a prognostic value of ^18^F-FMISO imaging, with a significant shorter OS in ^18^F-FMISO-positive lesions [53]. Further investigations are therefore needed to decipher the role of hypoxia imaging in the management of PDAC patients. 

### 2.4. ^68^Ga-FAPI

Cancer-associated fibroblasts (CAF) and extracellular fibrosis can contribute to up to 90% of the tumor mass [54]. CAFs differ from normal fibroblasts by their expression of fibroblast activation protein (FAP). FAP inhibitors (FAPI) were therefore developed as anti-cancer drugs and then as tumor-targeting radiotracers [55]. Several solid tumors, such as breast, lung, colorectal and PDAC cancers, were found to remarkably display high uptake of ^68^Ga-FAPI [56]. ^68^Ga-FAPI was found as a promising alternative to ^18^F-FDG in cancer patients. Studies of ^68^Ga-FAPI biodistribution demonstrated a high uptake in primary PDAC, lymph nodes and distant metastases, with low activity in healthy tissues. Indeed, a recent study revealed a higher tumor uptake of ^68^Ga-FAPI PET/CT as compared to ^18^F-FDG PET/CT in several cancers, including PDAC, as well as in more metastatic lesions [57]. In a study of 19 patients with primary or recurrent histologically confirmed PDAC, ^68^Ga-FAPI PET/CT showed a change in TNM stage in 10/19 (53%) patients compared to contrast-enhanced CT and a change in therapeutic management in 7 patients (37%) [58]. Recently, the tumor target volume for radiation therapy was more accurately defined with ^68^Ga-FAPI PET/CT as compared to the gold standard contrast-enhanced CT [59]. These preliminary results paved the way to conduct further investigations on the clinical value of ^68^Ga-FAPI in PDAC [60]. Moreover, the biodistribution of ^68^Ga-FAPI makes this tracer suitable for radioligand therapy.

**Figure 1:**
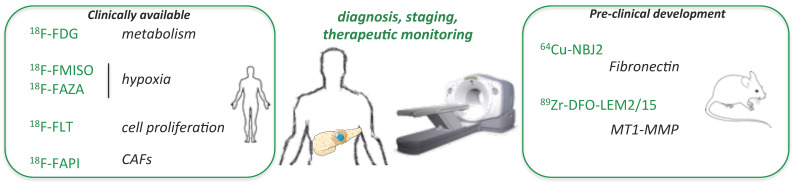
Radiotracers dedicated to diagnosis, staging and therapeutic monitoring of PDAC.

The different radiotracers evaluated in clinic for PDAC imaging are depicted in Figure 1.

## 3. Recent Developments in Nuclear Medicine for PDAC Imaging and Treatment

Currently, the ability to interrogate the target engagement of experimental therapies and the lack of specific imaging to assess therapy response are real concerns for clinical trials. The potential of nuclear medicine to non-invasively characterize tumors at the molecular level should be considered for the follow-up of patients with PDAC. Moreover, recent advances in the molecular mechanisms of PDAC tumorigenesis, and the advent of small-animal dedicated imaging systems have led to the development of new radiotracers. Indeed, several radiotracers dedicated for staging, monitoring or for the evaluation of patients’ eligibility for a targeted therapy (companion approach), or for targeted radionuclide therapy (theranostic approach), have been developed and evaluated in several PDAC mice models. Most of the radioisotopes described here are β+ emitters (^64^Cu, ^89^Zr and ^68^Ga) and therefore suitable for PET imaging. They can be potentially substituted on the vector molecule by β^-^ (^177^Lu and ^90^Y) or α-emitters (^225^Ac and ^213^Bi) isotopes for therapeutic use.

### 3.1. Radiotracers in Development Dedicated to Stroma Imaging

The extremely dense stroma of PDAC and its fundamental role during tumor progression gave the rationale for investigating the role of its components and the relevance of their targeting [61]. In addition to CAF targeting with the ^18^F-FAPI agent that could efficiently detect PDAC masses, radiotracers targeting fibronectin and matrix metalloproteinases (MMP) were recently developed for this application (Figure 1).

#### 3.1.1. Fibronectin-Targeting Agents

Fibronectin, a major component of the extracellular matrix, is overexpressed in PDAC samples and associated with advanced stages [62]. A single-domain antibody (sdAb) targeting the fibronectin (NJB2) was recently evaluated in mice models of breast cancer and PDAC [63]. ^64^Cu-NJB2 demonstrated high specificity for fibronectin-expressing lesions and exhibited a high tumor-to-background ratio at early time points. Moreover, ^64^Cu-NJB2 allowed early detection of PDAC as well as liver metastasis, whereas ^18^F-FDG did not. The biodistribution profile of sdAb, with limited uptake in liver and intestine, could overcome the known limitations of conventional mAbs to image metastasis with high contrast. The ability of ^64^Cu-NJB2 to detect PDAC and secondary masses remains to be evaluated in future clinical studies.

#### 3.1.2. MT1-MMP Imaging

The desmoplastic PDAC stroma contains many different proteases that play a key role in the crosstalk between cancer and stromal cells. MMPs are a family of zinc-dependent endopeptidases involved in the degradation of the ECM components. MMPs are considered as important contributors to PDAC progression [64]. Membrane-type 1 matrix metalloproteinase (MT1-MMP) upregulation promotes tumor progression and resistance to gemcitabine in a PDAC xenograft model [65]. MT1-MMP, as a candidate biomarker for non-invasive PDAC imaging, was evaluated using ^89^Zr-labeled antibodies (^89^Zr-DFO-LEM2/15). Despite liver uptake of the radiotracer, ^89^Zr-DFO-LEM2/15 allowed high-contrast imaging of PDAC in mice bearing orthotopically implanted, patient-derived xenograft tumors [66]. Clinical investigations of this radiotracer have not been conducted.

#### 3.1.3. CAFs

The tumor stroma represents an attractive target for the diagnosis but also for the delivery of therapeutic compounds. The absence of fibroblast-activation protein expression in normal tissue and the biodistribution profile of ^68^Ga-FAPI observed in the clinic offer a very encouraging opportunity for theranostic applications [67,68,69]. A proof of concept of radioligand therapy using ^90^Y-FAPI-04 in two patients with final-stage breast cancer was recently assessed [70]. ^90^Y-FAPI-04 led to a significant decrease in pain in these patients. In parallel, FAPI was labeled with ^225^Ac (^225^Ac-FAPI) and evaluated for its therapeutic potential in preclinical models of PDAC. ^225^Ac-FAPI treatment of PANC-1 xenografted mice showed a significant decrease in tumor growth in comparison to control mice [69]. However, ^225^Ac-FAPI therapeutic potential remains to be evaluated in clinical practice.

### 3.2. Radiotracers Targeting Tumor Antigen: Companion and Theranostic Approaches

**Figure 2:**
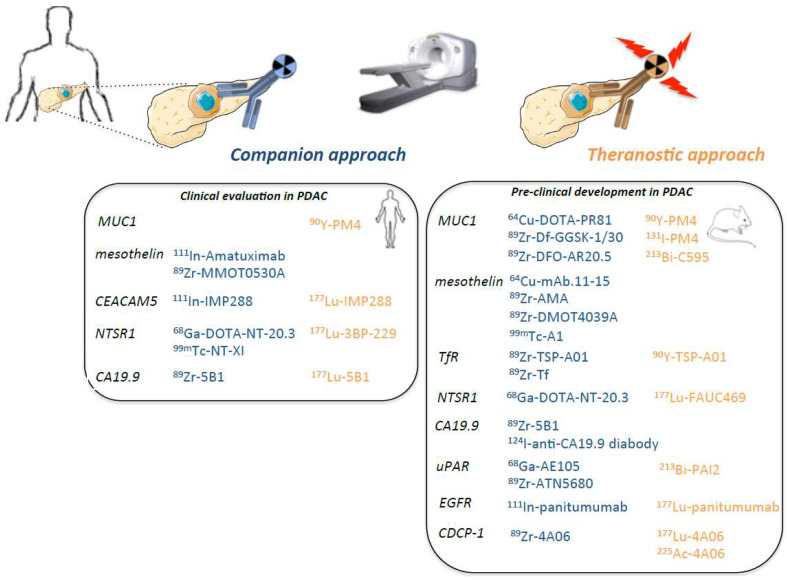
Radiotracers dedicated to companion and theranostic approaches currently in development.

The radiotracers dedicated to companion and theranostic approaches currently in development are presented in Figure 2.

#### 3.2.1. MUC1-Targeting 

Most of the tracers designed for the theranostic approach target the MUC1 protein. MUC1 is a 300–600 kDa membrane-bound mucin expressed in pancreatic ductal and acinar cells. More than 60% of PDAC overexpressed MUC1 and it has a pivotal role in PDAC progression [71,72]. In tumor cells, MUC1 binds to EGFR and β-catenin to enhance cell proliferation through the MAPK, Akt or Wnt/β-catenin pathways [73,74]. Moreover, MUC1 also induces epithelial–mesenchymal transition through activation of MMP13, Stat3 and PDGFR [71]. MUC1 also favors chemoresistance in PDAC by acting as a transcriptional regulator of multidrug-resistance genes [75]. MUC1 has therefore been linked to a poor prognosis in PDAC patients [76]. Some ^64^Cu- and ^89^Zr-radiolabelled monoclonal antibodies (mAbs) were recently developed for non-invasive measurement of MUC1-expression [77,78,79]. ^64^Cu-DOTA-PR81 and ^89^Zr-Df-GGSK-1/30 were successfully validated in MUC1-expressing breast cancers with a high ratio tumor/background uptake 72 h after injection [77,78]. More recently, ^89^Zr-DFO-AR20.5 was validated as a valuable tracer for the visualization of tumor tissues, including metastatic lesions in a pre-clinical model of ovarian cancer [79]. Nevertheless, all these radiotracers must be first validated for PDAC imaging and then clinically transferred for an eventual application.

Numerous MUC-1-targeting radiotracers dedicated to radioimmunotherapy of PDAC have been investigated in pre-clinical and clinical studies [80]. PAM4, an anti-MUC1 mAb, was radiolabeled with ^90^Y and ^131^I, and evaluated in combination with gemcitabine in pre-clinical models of PDAC. ^90^Y-PM4 provided greater growth inhibition as compared to ^131^I-PM4, with an improved median survival time (>26 weeks in the ^90^Y-PM4-treated group, and 17.5 weeks for the ^131^I-PM4-treated group) [81]. Gold et al. further showed that a combination of ^90^Y-PM4 with gemcitabine enhanced tumor response in mice models of PDAC [82]. This observation led to the clinical evaluation of ^90^Y-PM4 in PDAC patients. ^90^Y-PM4 treatment was well tolerated, with manageable hematologic toxicity [83]. Low-dose gemcitabine in combination with ^90^Y-PM4 was evaluated in 38 advanced PDAC patients. Partial response and stable disease were found in 16% and 58% of patients, respectively [84]. Phase III was recently conducted in 334 PDAC patients but failed to demonstrate any significant improvement of OS when compared to the placebo (NCT01956812). The superior therapeutic efficacy of alpha-therapy in comparison to beta-therapy gave rise to the development of alpha-emitter radiotracers [85]. ^213^Bi-C595, a monoclonal antibody, was cytotoxic in vitro on PDAC cells and in vivo on a model of ovarian cancer [86,87]. Alpha-therapy remains to be evaluated in PDAC. 

#### 3.2.2. Mesothelin-Targeting with Antibody-Derived Radiotracers

Mesothelin is a 40-kDa, GPI-anchored membrane protein whose expression is very low in normal tissues (pleura, peritoneum and pericardium). However, mesothelin is overexpressed in several solid tumors, including in 80 to 85% of PDAC [88,89]. Mesothelin is a key player in sustaining cell proliferation and invasion of PDAC [89]. The limited expression of mesothelin in normal tissues as well as its overexpression in a broad range of tumors makes it an attractive and promising target for therapy. Several anti-mesothelin targeting drugs, including an antibody-based approach, immunotoxins or CAR-T cells, are under clinical investigations [90,91]. Some radiotracers serving as companion markers of anti-mesothelin therapies are currently in development. Some mAbs have been evaluated for PDAC targeting in pre-clinical mouse models and in patients. Specific signals in mesothelin-positive lesions were detected using ^64^Cu- and ^89^Zr-radiolabeled mAbs [92,93,94]. Clinical investigations showed high ability of ^111^In- and ^89^Zr-radiolabeled mAbs for the phenotypic imaging of mesothelin-expressing PDAC [95,96]. Nevertheless, hepatic elimination of mAbs and their slow blood clearance constitute important limitations. Single-chain variant (ScFv) and single-domain antibodies displaying optimal pharmacokinetics have been validated in pre-clinical studies and will thus deserve to be fully considered for clinical development [89,97,98]. 

In addition to their potential to serve as a companion marker for the selection of patients eligible for anti-mesothelin therapies, anti-mesothelin mAbs have been radiolabeled with alpha-emitter particles for targeted radionuclides therapy. A 227-thorium (^227^Th)-radiolabeled anti-mesothelin mAbs has recently been validated in pre-clinical models of ovarian cancer [99]. A Phase I trial using this compound is ongoing in patients with locally advanced or metastatic PDAC (NCT03507452).

#### 3.2.3. Transferrin Receptor (TfR)-Targeting Agents

Transferrin receptor (TfR) is a type II transmembrane heterodimeric glycoprotein involved in iron uptake. Tumor cells are highly dependent on iron for proliferation when compared to normal cells and are more sensitive to iron deprivation [100,101]. TfR is therefore upregulated in malignant cells [102], including PDAC [97]. More generally, TfR is a key actor of several hallmarks of cancer, including cancer cell proliferation, migration and invasion [103]. TfR have consequently emerged as a candidate for antibody-mediated cancer therapy [104]. Different antibody-based strategies that target TfR in malignant cells have been developed and assessed in pre-clinical models of solid tumors. This includes in particular mAbs that can be combined to therapeutic agents (chemotherapy), toxins or radioisotopes, or ScFv [105]. Indeed, the internalization of TfR ligands opens unique opportunities for the delivery of toxic agents into malignant cells. Among the therapies, the combination of SGT-53 (nanocomplex carrying the p53 gene) with docetaxel was evaluated in 12 patients (1 PDAC) in a Phase 1b dose-escalation trial. The RECIST partial response observed in 3/12 patients and the stable disease observed in 2 patients support the Phase 2 testing this combination [106]. A Phase II study of the combination of SGT-53 and gemcitabine/nab-paclitaxel is ongoing in patients with metastatic PDAC (NCT02340117). ^89^Zr-TSP-A01, an anti-TfR mAb, was successfully evaluated in MiaPaCa-2-bearing mice for its ability to bind to TfR-positive tumors [107]. This antibody was also radiolabeled with ^90^Y (^90^Y-TSP-A01) and considered for radioimmunotherapy. One injection of ^90^Y-TSP-A01 in tumor-bearing mice (MiaPaCa-2-derived tumors) resulted in an almost complete disappearance of tumors, making it a very promising agent [108]. Nevertheless, the clinical potential of such a theranostic agent remains to be further investigated. More recently, transferrin was radiolabeled with ^89^Zr and evaluated for its ability to target PDAC. 

Moreover, ^89^Zr-transferrin was evaluated for its potential to non-invasively assess the signaling downstream KRAS to stratify patients that will benefit from targeted therapies. ^89^Zr-transferrin was confirmed to be a valuable tool to evaluate oncogene status, as TfR is a downstream target of the KRAS pathway, and the target engagement of MYC- and ERK-targeted therapies [109,110].

#### 3.2.4. CEACAM5-Targeting Agents

CEA, also called carcinoembryonic antigen–related cell adhesion molecule 5 (CEACAM5), is a 200-kDa protein belonging to the CEACAM family and anchored to the cell surface by a glycosylphosphatidylinositol (GPI). Despite poor sensitivity and specificity, serum CEACAM5 is a prognostic biomarker of PDAC [111]. Soluble CEACAM5 is related to tumor burden in patients with PDAC, thereby giving the rationale for its use in clinical practice [112]. Histological analysis of tumor samples revealed its ability to identify viable tumor cells before and after neoadjuvant treatment [113]. Moreover, the high tumor-to-normal ratio of CEACAM5 expression supports the development of anti-CEACAM5 imaging agents for diagnosis and staging of PDAC. That said, such potential need to be further investigated in PDAC. 

Of note, the theranostic potential of anti-CEACAM5 radiolabeled antibodies was supported by a clinical pilot study carried out in five patients having metastatic colorectal cancer (mCRC) [114]. ^111^In-IMP288—a bispecific, engineered antibody—rapidly and selectively accumulated in tumors with a high tumor-to-tissue ratio. Administration of ^177^Lu-IMP288 therapeutic antibody was well tolerated without any observed adverse effect. The therapeutic potential of such an antibody remains to be demonstrated and evaluated in PDAC patients. A Phase II carried out in mCRC patients is investigating the ability of another mAb (^124^I-M5A) to detect liver metastasis.

#### 3.2.5. Neurotensin Receptor-Targeting Agents

Neurotensin (NTS) is a physiological hormone, which affects the function of the gastro-intestinal tract through its cellular receptor (NTSR1). In 1998, both NTS and NTSR1 were reported to be more expressed in PDAC tissues when compared to normal samples [115]. Expression of NTSR1 correlated with histological grade and higher expression of NTSR1 was detected in patients with liver metastasis [116,117]. The signaling pathway of NTS/NTSR1 is well known in cancer—NTS/NTSR1 triggers PKC, PI3K and ERK1/2 activation, leading to aberrant survival and proliferation of tumor cells [118]. NTS/NTSR1 also activated Rho GTPases and the focal adhesion kinase, key players involved in cell migration phenomena [119,120,121]. The selective inhibition of NTSR1 in mice attenuated the tumorigenicity of the pancreatic cells [122]. Based on the selective expression of NTS/NTSR1 and its role in PDAC progression, several NTSR1-targeting agents were proposed for non-invasive imaging, treatment monitoring tools and vectorized radiotherapy [123]. ^68^Ga-DOTA-NT-20.3, an analogue of NTS that displays a high affinity for PDAC cells, was evaluated in orthotopic xenograft models of PDAC [124,125]. ^68^Ga-DOTA-NT-20.3 exhibited high tumor uptake and high contrast between the tumor and background, at early time points with a fast blood clearance. This promising result led to a recent first clinical trial in humans, where the safety, tolerability and ^68^Ga-DOTA-NT-20.3 uptake in PDAC (n = 3) were observed [126]. However, future investigations need to be assessed in a larger cohort of patients. Another NTS radiolabeled-analogue (^99m^Tc-NT-XI) was previously evaluated in four PDAC patients. Despite the small number of patients, the relevance of the NTSR phenotypic imaging method has been reported [127]. A PET-dedicated radiotracer was recently developed and evaluated in prostate tumor-bearing mice. ^64^Cu-DOTA-NT specifically binds NTSR-expressing tumors [128]. The biochemical structure of DOTA also allows ^177^Lu radiolabeling, which is dedicated to targeted radiotherapy.

Twenty years ago, one of the first NTS analogues was radiolabeled with ^188^Re and evaluated in a radiotherapy study using HT29 colon cancer-bearing nude mice [129]. Three injections of ^188^Re-NT-XIX led to a 50% decrease in tumor growth. Only few studies have reported the pre-clinical efficacy of ^177^Lu-radiolabelled NTS in PDAC. ^177^Lu-FAUC469, a triazolyl-linked DOTA-derivative, was shown to display high tumor growth inhibition in PDAC-bearing mice [123]. The first patient injection of ^177^Lu-labelled DOTA-conjugate of NTS (^177^Lu-3BP-227) was performed in six PDAC patients [130]. ^177^Lu-3BP-227 was well tolerated and one patient achieved a partial response and experienced an improved quality of life. Despite this observation, which provides clinical evidence of ^177^Lu-3BP-227 treatment feasibility, it remains to be further evaluated in a Phase II study.

#### 3.2.6. CA19.9-Targeting Agents

Serum carbohydrate antigen 19.9 (CA19.9) is regularly measured in patients with PDAC [131]. The diagnostic potential of soluble CA19.9 was evaluated in several studies. CA19.9 levels are upregulated up to 2 years prior to a PDAC diagnosis, and its level correlated with tumor stage [132,133,134]. However, CA19.9 as a soluble biomarker has limitations, including a low positive predictive value, and augmented values in pancreatico-biliary diseases [135]. Nonetheless, CA19.9 remains an attractive target for specific diagnosis and therapy as it is expressed at high levels at the surface of cancer cells. ^89^Zr-5B1, an anti-CA19.9 radiolabeled antibody, allowed non-invasive imaging of PDAC xenografts with an intense uptake (>100% of injected activity per gram of tissue), and notably a low activity in the liver and spleen [136,137]. Moreover, when unlabeled 5B1 was administered prior to ^89^Zr-5B1, the radiotracer significantly enhanced the image contrast and tumor-to-tissue ratio [137]. Due to the potential of ^89^Zr-5B1 to non-invasively measure CA19.9 expression, a dose-escalation trial of ^89^Zr-5B1 in PDAC patients was performed. This dose-escalation study confirmed these promising results in 12 patients with metastatic CA19.9-expressing tumors [138]. Anti-CA19.9 diabodies were also engineered and provided specific molecular imaging in tumor xenograft models [139,140].

The biodistribution profile of 5B1 validated the rationale for its use as a theranostic agent. A Phase I study on ^177^Lu-5B1 therapy was initiated and is ongoing (NCT02672917). The aim of this study is to determine the maximum tolerated dose of ^177^Lu-5B1, as well as its pharmacokinetics and dosimetry characteristics.

#### 3.2.7. uPA/uPAR System

The tumor cell-associated urokinase plasminogen activator (uPA) system, including the serine protease uPA, its membrane-associated receptor uPAR and the plasminogen activator inhibitor (PAI2), play a crucial role in cell proliferation and metastasis [141,142]. uPAR binds uPA to convert inactive plasminogen to active plasmin, disrupting the ECM, and allowing invasion and metastatic spread of malignant cells [143]. Aberrant expression of the uPA-uPAR system components has been detected in a wide range of tumors [144]. Around 60% of patients with PDAC express uPAR in neoplastic or stromal cells [145]. Increased gene expression is associated with poor survival in PDAC patients [146]. Chen et al. showed that uPAR expression could discriminate between PDAC and pancreatitis [147]. Much attention has been deployed in the development of non-invasive imaging of uPA systems. The peptide antagonist AE105 and the anti-uPAR mAb ATN-658 were developed and radiolabeled with ^68^Ga (^68^Ga-AE105) and ^89^Zr (^89^Zr-ATN-658), respectively [148,149,150]. As a 1-kDa molecule, ^68^Ga-AE105 exhibited rapid blood clearance, thereby allowing tumor imaging at early time points with a good contrast. ^89^Zr-ATN-658 has a much longer half-life in the blood, offering then a possibility of acquisition at later time points. ^68^Ga-AE105 was evaluated in a Phase I study in patients with prostate, breast and bladder cancers [150,151]. The administration of ^68^Ga-AE105 was well tolerated and primary tumor uptake could be evidenced with high contrast 60 min post-injection. Moreover, the capacity of this tracer to accurately detect lymph nodes metastasis makes it suitable to allow the staging of solid tumors. To date, no evaluation in PDAC patients was performed but ^177^Lu-AE105 was evaluated in a disseminated metastatic prostate cancer model [152]. uPAR-targeted radionuclide therapy significantly reduced the number of metastatic lesions when compared to the vehicle and non-targeted ^177^Lu-groups. Targeted alpha-therapy was evaluated in a xenograft model of PDAC using the ^213^Bi-PAI2 radiotracer, and a significant tumor growth inhibition could then be evidenced [153]. However, further investigations are needed for PDAC and should ultimately allow a better overall understanding. 

#### 3.2.8. EGFR

Epidermal growth factor receptor (EGFR) is a transmembrane tyrosine kinase receptor involved in the regulation of cell proliferation, survival and apoptosis [154]. Overexpression of EGFR was reported in a wide range of tumors, including breast, colon or lung cancers [154,155]. For PDAC, the EGFR levels ranged from 40 to 70% and its expression is associated with advanced stages and poor clinical outcomes [156,157,158,159,160]. In addition, EGFR signaling contributes to the transition of normal pancreatic epithelia to neoplasms [161]. Two classes of inhibitors have been developed for the EGFR-targeting: tyrosine kinase inhibitors (erlotinib) and EGFR mAbs (cetuximab and panitumumab). The Food and Drug Administration approved the combinatory use of erlotinib with gemcitabine as a first-line therapy for patients with advanced PDAC, since a significant improvement in OS was evidenced in comparison to gemcitabine treatment alone [162]. The measure of EGFR expression with SPECT and TEP imaging was performed using sdAbs radiolabeled with ^99m^Tc- or ^68^Ga [163,164,165,166]. ^99m^Tc-7C12 sdAb represents a valuable tool for monitoring erlotinib response in mice models of epidermoid carcinoma [166]. ^89^Zr-DFO-ZEGFR:2377, a radiolabeled affibody was also validated as a non-invasive tool to quantity EGFR expression in this tumor model [167]. ^89^Zr-cetuximab was evaluated in 10 patients with metastatic colorectal cancers and treated with cetuximab. Despite the low number of patients, a correlation between ^89^Zr-cetuximab uptake in secondary lesions and tumor response could be demonstrated [168]. The theranostic couple ^111^In- and ^177^Lu-panitumumab was recently evaluated for EGFR imaging in mice models of PDAC [169]. The radio-immunotherapy was further evaluated in PANC-1 xenografts. ^177^Lu-panitumumab strongly impedes tumor growth without any significant detected side effect (blood counts, serum alanine aminotransferase and creatinine) [170]. To date, no investigation of radioimmunotherapy targeting EGFR has been evaluated in a clinical study.

#### 3.2.9. CDCP-1

The CUB domain-containing protein 1 (CDCP1) is a glycosylated transmembrane protein discovered 20 years ago by Scherl-Mostageer [171]. CDCP1 expression is associated with the loss of anchorage in epithelial cells during mitosis [172]. CDCP1 regulates the SRC/PKC, PI3K/Akt and RAS/ERK pathway, making it a player located at the nexus of tumorigenesis and metastasis processes [173]. Its expression has therefore been associated with a poor prognosis in several cancers, including PDAC [174,175]. These findings have stimulated the development of CDCP1-targeting agents for detection and treatment of cancers. Small molecules (Pd-Oqn) or antibodies (10D7) targeting CDCP1 have been developed and evaluated in pre-clinical studies. Peritoneal injection of Pd-Oqn in mice models was associated with a significant decrease in the peritoneal dissemination of gastric tumors [176]. The combination of 10D7 with monomethyl auristatin E cytotoxin (10D7-MMAE) reduced tumor burden in mice models of metastatic ovarian cancers [177]. 10D7 was radiolabeled with ^89^Zr (^89^Zr-10D7) and evaluated in an ovarian cancer model and a high tumor-to-background ratio could be obtained 144 h following administration [177]. Moreover, peritoneal masses were readily observable using ^89^Zr-10D7. Of note, ^89^Zr-10D7 was also found to detect PDAC tumor-derived xenografts [178]. Another antibody, 4A06, was recently radiolabeled with ^89^Zr (^89^Zr-4A06) for PET imaging of PDAC tumors but also with ^177^Lu- and ^225^Ac-, respectively, for β^-^- and α-emitter-targeted therapy [179]. ^89^Zr-4A06 detected CDCP-1 expressing PDAC (seven cell lines). Treatment with a single dose of ^177^Lu-4A06 significantly reduced tumor volumes in comparison to the control. Despite significant results when compared to the control conditions, the ^225^Ac-4A06 effect was less pronounced as compared to ^177^Lu-4A06. Similarly, further investigations deserve to be conducted for the clinical evaluation of these compounds. 

## 4. Conclusions 

Despite intensive efforts in the field of pancreatic cancer research, early-stage detection, treatment response monitoring and development of efficient therapeutic strategies in PDAC are still in urgent need. The field of nuclear medicine, for its ability to image a molecular-target and to treat tumors, could represent a way to improve the management of these patients. The upcoming availability of ^68^Ga-FAPI, with its ability to discriminate inflammation from neoplastic masses, to allow an accurate staging of patients, favors its future use in clinical routines. ^68^Ga-FAPI seems to overcome the known limitations of other available imaging modalities. Nonetheless, the clinical value of ^68^Ga-FAPI in PDAC remains to be further investigated.

The advent of theranostic and radioligand therapy in nuclear medicine has opened new opportunities for the management of solid tumors, including PDAC. More than 15 agents have been developed for the radioligand therapy of PDAC, including β- (^177^Lu and ^90^Y) and α-emitter (^225^Ac and ^213^Bi)-radiolabeled compounds. All these theranostic agents exhibit a high ability to non-invasively image PDAC with a high tumor-to-background ratio and strong anti-tumor efficacy in pre-clinical models of PDAC. Although the clinical applicability (toxicity and objective tumor response) of most of these agents remains to be further evaluated, they undoubtedly have great potential to be integrated into combination anti-cancer therapies.

## Abbreviations

CAF	Cancer-associated fibroblast
CA19.9	Carbohydrate antigen19.9
CDCP1	CUB domain-containing protein1
CEACAM5	Carcinoembryonic antigen-related cell adhesion
EGFR	Epidermoid growth factor receptor
FAP	Fibroblast activating protein
FAPI	Fibroblast activating protein inhibitor
FAZA	Fluoroazomycin arabinoside
FDG	Fluorodeoxyglucose
FLT	Fluorothymidine
FMISO	Fluoromisonidazole
mAb	Monoclonal antibody
MMP	Matrix metalloproteinase
MRI	Magnetic resonance imaging
MUC1	Mucin-1
NTS	Neurotensin
OS	Overall survival
PAI2	Plasminogen activator inhibitor-2
PDAC	Pancreatic ductal adenocarcinoma
PET	Positron emission tomography
PFS	Progression-free survival
SdAb	Single-domain antibody
SPECT	Single-photon emission computed tomography
SUV	Standardized uptake value
TfR	Transferrin receptor
uPa	Urokinase-type plasminogen activator
